# The Role of Artificial Intelligence in Biomaterials Science: A Review

**DOI:** 10.3390/polym17192668

**Published:** 2025-10-02

**Authors:** Andrea Martelli, Devis Bellucci, Valeria Cannillo

**Affiliations:** Department of Engineering “Enzo Ferrari”, University of Modena and Reggio Emilia, Via Vivarelli, 10, 41125 Modena, Italy; andrea.martelli@unimore.it (A.M.); valeria.cannillo@unimore.it (V.C.)

**Keywords:** artificial intelligence, biomaterials, materials, data science, machine learning

## Abstract

Biomaterials can be defined as materials that interact positively with living tissues, restoring compromised functions, or enhancing tissue regeneration. Currently, biomaterial research often relies on a “trial-and-error method”, involving numerous experiments driven largely by experience. This strategy leads to a substantial waste of resources, such as manpower, time, materials, and finances. Optimizing the process is therefore essential. A recent and promising approach to this challenge involves artificial intelligence (AI), as demonstrated by the growing number of studies in this field. AI algorithms rely on data and empower computers with decision-making capabilities, mimicking aspects of the human mind and solving complex tasks with little to no human intervention. Due to their potential, AI and its derivatives are now widely used both in everyday life and in scientific research. In biomaterials science, AI models enable data analysis, pattern recognition, and property prediction. The aim of this review article is to highlight the key results achieved through the application of AI in the field of polymers for biomedical applications and, more broadly, in the development of advanced biomaterials. An overview will be provided on how an AI algorithm works, the differences between traditional programming and AI-based approaches, and their main limitations. Finally, the core topic will be addressed by categorizing biomaterials according to material class.

## 1. Introduction

Artificial intelligence (AI) can be defined as the capacity of machines to emulate human cognition, make decisions, and perform complex reasoning through advanced algorithms [1]. Its applications are rapidly expanding, from everyday tools such as smartphone virtual assistants to transformative innovations in scientific research. In medicine, for instance, AI has been employed to identify carcinogenic human metabolites, detect skin cancer, and support the diagnosis of neurodegenerative disorders such as Parkinson’s disease [2,3,4]. Likewise, AI holds great promise for advancing research in the field of biomaterials.

Biomaterials science is a multidisciplinary field characterized by the symbiotic interplay between various disciplines such as materials science, biology, and biomechanics [5]. Within this context, a biomaterial is any material for biomedical use, which can find applications, for example, in the creation of a prosthesis or implant, aiding in the recovery of a function of the body that is totally or partially compromised. An implantable biomaterial can interact positively with the biological environment of the human body and can be used for both partial and full tissue replacement. Additionally, biomaterials are used in the field of tissue engineering to produce scaffolds, i.e., three-dimensional porous structures onto which the patient’s cells are seeded. These cells then generate new tissue after appropriate maturation within a bioreactor [6,7]. In this case, the advent of additive manufacturing (AM) and, in particular, 3D bioprinting, with its simultaneous deposition of materials and cells, has revolutionized the production of scaffolds for tissue regeneration. Indeed, this technology enables precise customization of scaffolds, enhancing tissue integration and reproduction, while controlled cell distribution replicates natural architecture, facilitating healing. Moreover, scaffolds can release growth factors, speeding up tissue regeneration [8,9,10].

Among biomaterials, examples can be found belonging to all traditional classes of materials, including metals, ceramics, glasses, polymers, and composites, as well as materials of natural origin, such as collagen, hyaluronic acid, or alginate. The choice of material depends on the specific application. Metals are often selected for their exceptional mechanical strength, making them ideal for load-bearing applications such as joint replacements. Additionally, certain alloys exhibit shape memory properties, which are particularly useful for stent fabrication [11]. Ceramics are characterized by high stiffness, hardness, and wear resistance, along with good to excellent biocompatibility. For these reasons, ceramics are commonly used in orthopaedic, dentistry, and tissue engineering applications [12,13]. Polymers, both natural and synthetic, offer high tuneable mechanical and biological properties. They can also be easily shaped, making them suitable for a wide range of applications, from suture threads to tissue engineering [14,15]. Composites, due to their hybrid nature, can combine key properties from different material classes, enabling the development of new features, such as controlled degradation behaviour. Thanks to their versatility, composites are used in numerous applications [16,17].

Currently, biomaterials research largely depends on a “trial-and-error” approach, which frequently results in substantial waste of resources, including personnel, time, materials, and funding [18]. A potential solution to this problem is the use of AI for pattern recognition and property prediction, with a consequent reduction in extensive experimentation.

Although several reviews have discussed the application of AI in biomaterials, they typically focus on a single material class or provide only a broad overview of AI techniques [19,20,21,22]. To address this gap, the aim of this review is to highlight the key results achieved through the application of AI models in biomaterials science. The first part will explain how AI models operate and discuss the main challenges associated with their use. Subsequently, the major findings in biomaterials research will be presented, organized by material class.

## 2. Methodology

**Purpose:** This review aims to highlight recent advances, identify current challenges, and outline future perspectives for the integration of AI in biomaterials science. Given its exploratory and integrative objectives, it adopts a narrative review format rather than a systematic review.

**Scope:** The review is limited to peer-reviewed publications in English reporting AI or machine learning applications in materials science, with particular emphasis on biomedical uses. Non-peer-reviewed sources were excluded. Literature was retrieved from major databases (e.g., Scopus, Google Scholar, and PubMed) using keyword combinations such as *Artificial Intelligence*, *Machine Learning*, *Biomaterial*, and *Polymer*.

**Function:** The review organizes and critically synthesizes the retrieved studies by material class (polymers, ceramics, metals, composites) and AI function (property prediction, process optimization, material design, or biological response prediction), enabling balanced comparisons across different systems and algorithms.

**Intent:** The intention is to provide researchers and clinicians—including those not specialized in AI—with an integrated overview that identifies knowledge gaps, highlights promising approaches, and offers guidance for future experimental and regulatory developments.

## 3. Artificial Intelligence: From Data Science to Machine Learning

AI can be defined as a system capable of mimicking human cognition by making decisions and processing complex reasoning through advanced algorithms, with little to no human intervention [1,23]. To achieve this, AI relies heavily on data science, which is one of its key components. Data science involves extracting knowledge from large datasets, both structured and unstructured. This data-driven approach enables AI to make predictions about future outcomes, with accuracy depending on the size and quality of the dataset. Initially, AI was based on hard-coded rules linked by logical inference, such as “if the premises are true, then the conclusion is...”. As a result, early AI systems were limited by the human ability to express all the knowledge required to perform complex tasks [24].

Machine learning (ML), with its approach based on data science, overcomes these limitations. ML improves its accuracy through an iterative process, learning from training data and discovering new patterns not explicitly programmed in each cycle [1]. A major advantage of the ML approach is its ability to address both forward and inverse problem designs. In simpler terms, this means that ML can be used for predictive tasks, such as forecasting the properties of a given material structure. Additionally, it can be applied to inverse tasks, such as identifying which materials exhibit specific properties based on predefined requirements.

ML is broadly categorized into four types: supervised, unsupervised, semi-supervised, and reinforcement learning.

Supervised learning relies on labelled data and user-defined output targets, enabling the machine to learn from these labelled examples. The goal is to learn a mapping function from input to output. For instance, labelled data might consist of thousands of images of materials, each annotated with information about the material type. The resulting model will be able to distinguish one material from another.

In contrast, unsupervised learning works on unlabelled data. In this case, the goal is to discover hidden patterns or clusters in the data without explicit guidance on what to look for [25]. For example, given a dataset of mechanical properties associated with unknown materials, this approach enables ML to identify patterns and potentially cluster the materials into conventional categories such as metals, ceramics, polymers, and composites.

Semi-supervised learning combines aspects of both supervised and unsupervised learning by leveraging both labelled and unlabelled data. The aim is to improve prediction accuracy beyond what can be achieved using only labelled data [26].

Reinforcement learning, on the other hand, is an approach based on feedback from the environment, in which algorithms tackle sequential decision-making problems in dynamic and often uncertain settings [27].

The objective of any reinforcement learning algorithm is to learn the rules that connect its actions with the environment, thereby achieving rewards for favourable outcomes. This means that algorithms select their actions based solely on their current observations, and by the end of the learning process, they are able to choose actions that yield optimal outcomes and higher rewards [28,29]. In other words, reinforcement learning follows a computational trial-and-error strategy. A practical example of this approach is an optimization process in which the model maximizes desired material properties while minimizing costs or waste.

Within supervised and unsupervised learning, various models exist, each tailored to specific tasks.

Classification algorithms group input data into specific categories based on their features and are typically used with labelled data to predict class labels. Regression models, on the other hand, predict numerical values from input features, requiring labelled data to learn the mapping between inputs and desired numerical outputs [30]. Unlike classifiers, which produce discrete outputs, regression models can predict continuous values [31]. Both classification and regression models are commonly used for prediction tasks within supervised learning. Association learning aims to discover meaningful relationships between variables in unlabelled datasets, identifying associations and correlations that occur more frequently than would be expected by chance [26]. Clustering, often used with unlabelled datasets, groups similar entities based on quantitative similarity measures [26]. Association learning and clustering are commonly used for recognizing underlying structures and patterns in data within an unsupervised learning approach [1].

These models use various methods—such as decision trees, random forests, and linear regression—that differ in prediction accuracy, training speed, and the number of variables they can handle. Therefore, selecting the appropriate algorithm is essential and should be based on the specific problem being addressed.

A further evolution and subcategory of ML is deep learning (DL), which can tackle more complex tasks through a specialized approach [24]. One significant advantage of DL is its ability to extract higher-level features from raw input data, a process that could be time-consuming, fragile, and not easily scalable with traditional ML methods. However, it is important to note that employing a reliable DL model typically necessitates a larger number of samples compared to other ML models [32]. 

DL relies on multi-layer artificial neural networks (ANNs), also known as deep neural networks. These networks are inspired by the biological nervous system and consist of interconnected elements called neurons. Neurons are arranged in layers, and each layer analyses different aspects of the input data through previously presented algorithms. For example, the first layer might detect the colour of an image, the next one might identify shapes, and subsequent layers gradually build up to recognize the entire object. In this way, the input signal propagates through each layer, allowing the model to train and refine its predictions [33,34]. 

**Figure 1:**
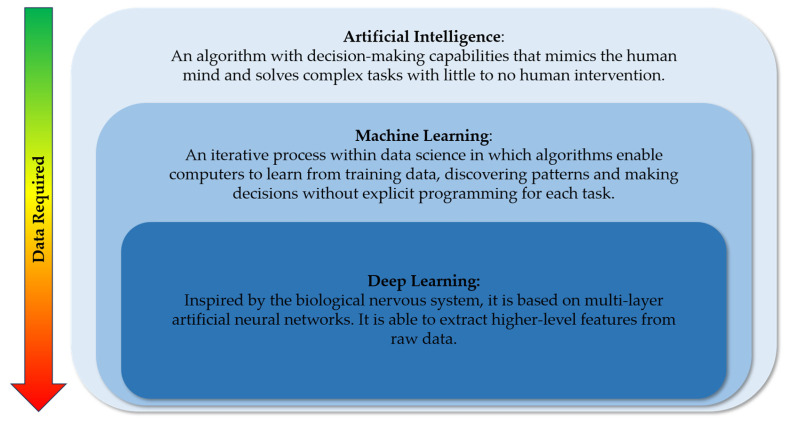
Representation of the relationships among AI, ML, and DL, with increasing data requirements.

**Figure 2:**
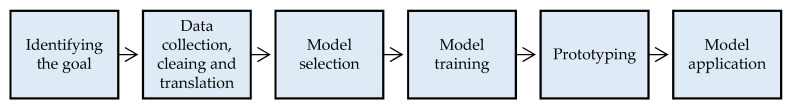
The AI approach, step by step.

For a schematic representation of the relationships among AI, ML, and DL, please refer to Figure 1. To provide an accessible overview of the AI approach, we will present a simplified version of the algorithm in Figure 2. For a more comprehensive understanding of the process, please refer to the cited reviews [35,36]. For practical, hands-on guidance on developing AI, readers can refer to the work of Meyer et al. [37].

The process begins by identifying a specific goal, typically related to material or process design, or knowledge discovery. This is followed by data collection, which may involve conducting experiments or simulations and extracting information from reference materials. It is essential to emphasize that data quality is paramount, necessitating a data-cleaning step. Once the data is prepared, it needs to be formatted for the AI algorithm, often involving the conversion of non-numerical information into a machine-readable format. Data collection and translation are particularly critical in material science and will thus be the focus of the following sections. 

The next critical step involves selecting an appropriate model based on the specific problem at hand. This choice depends on various factors, such as task type (e.g., property prediction, inverse design), the size and type of data (e.g., numerical, graphical), and the requirements for interpretability. Once the model is chosen, it needs to be trained, which involves the optimization of its parameters for best performance. Prototyping at this stage is crucial, as it allows for assessing the reasonableness of the results. Common challenges include model overfitting and underfitting. Overfitting occurs when a model captures unnecessary details from the training dataset, which can degrade its performance. To mitigate this, techniques such as resampling (e.g., dropout) or regularization (e.g., Lasso or Ridge regression) can be applied, or a portion of the training data can be set aside for validation. Underfitting, on the other hand, happens when the model fails to capture underlying patterns due to insufficient data, resulting in poor performance. In such cases, the primary solution is to increase the amount of training data.

To determine which model or dataset best meets the study’s objectives, different models can be compared using evaluation metrics such as *R*^2^ or the F1 score [38]. R^2^ and F1 are performance metrics representing, respectively, the proportion of variance in the dependent variable explained by the independent variables, and the harmonic mean of precision and recall. Once a model has been successfully trained and validated through prototyping, it is ready for its intended application, contributing to the achievement of the initial goal. By systematically following these steps, AI methods can be effectively applied to address the specific problem at hand.

It is evident that building a traditional algorithm differs significantly from creating an AI algorithm. Traditional algorithms are rule-based procedures designed to solve specific computational problems, which limits their versatility without human intervention. In contrast, AI algorithms learn from data without explicit inputs, thus expanding their versatility and ability to discern patterns as they are free from a priori assumptions. This suggests that traditional algorithms rely heavily on human input, while AI algorithms are highly dependent on data, which must be both numerous and high-quality. Furthermore, traditional algorithms are fully programmed and can be considered “white-box”, unlike AI algorithms, which are regarded as “black-box”, resulting in a lack of interpretability in the decision-making process. Finally, AI algorithms typically require more advanced hardware compared to traditional algorithms [39,40,41,42]. The advantages and disadvantages of both methods are summarized in Table 1.

### 3.1. Data Collection

As previously mentioned, AI operates using a data-driven approach. A key challenge in crafting robust AI algorithms lies in the availability and comparability of data [43]. For well-known properties (e.g., thermal characteristics) it is possible to refer to handbooks or online databases (Table 2) to increase the size of datasets. However, experimental datasets frequently encounter constraints and lack comparability, primarily due to variations in experimental procedures or data analysis methods. Indeed, although numerous handbooks and databases exist for different materials, they often suffer from fragmented data and provide only a limited range of properties.

This poses a significant challenge when attempting to predict poorly labelled or sparsely measured properties, such as the in vivo degradation of biomaterials, due to the limited size of available datasets [44]. Furthermore, the lack of a clearly defined and measurable standard for concepts like biocompatibility further limits the effectiveness of data mining techniques, as highlighted by Mateu-Sanz et al. [45].

**Table 2:** Examples of online databases for material properties.

Database	Material	Reference
PI1M	Polymer	[46]
PolyInfo	Polymer	[47]
Khazana	Polymer	[48]
Polymers: a Property Database	Polymer	[49]
Polymer Property Predictor and Database	Polymer	[50]
Block Copolymer Phase Behavior Database	Polymer	[51]
CAMPUS	Polymer	[52]
Electron Affinity and Ionization Potential Data	Polymer	[53]
Silkome	Spider silk	[54]
SciGlass	Ceramic	[55]
Interglad	Ceramic	[56]
The Materials Project	Miscellanea	[57]
Ansys Granta	Miscellanea	[58]
MatWeb	Miscellanea	[59]
Atomly	Miscellanea	[60]

Small datasets can lead to problems such as model underfitting or overfitting, and several examples of this can be found in the following paragraphs [61,62,63,64,65]. In such cases, two main strategies can be employed to address the issue: increasing the dataset size during data collection, or selecting AI models able to perform well with limited amounts of data. Examples of such models include support vector machines (SVM), Gaussian process regression (GPR), deep neural networks (DNN) or generative models like generative adversarial networks (GAN), variational autoencoders (VAE) [66,67].

One effective strategy to address the challenge of limited data is to leverage *simulated datasets* derived from well-understood properties, as exemplified by John et al. in their research on optoelectronic properties [68]. This approach shows great potential, particularly for generating labelled datasets for properties that are rarely characterized due to their inherent complexity or limited application. However, it is important to recognize that error propagation represents a significant drawback of this method. Therefore, careful consideration and thorough evaluation are essential to ensure the accuracy and reliability of the predicted properties, thereby safeguarding their fidelity.

As an alternative, experimental data can also be leveraged. However, as mentioned earlier, the trial-and-error approach consumes significant time and resources. To mitigate this, high throughput or automated experiments can be employed to acquire more data in a shorter timeframe, optimizing the efficiency of the data generation process [69,70]. Strategies for high-throughput experimentation to create polymer libraries were comprehensively outlined by Oliver et al. [71]. In general, high-throughput approaches hold promise for properties that can be measured using small quantities of material, such as cytotoxicity [44].

**Figure 3:**
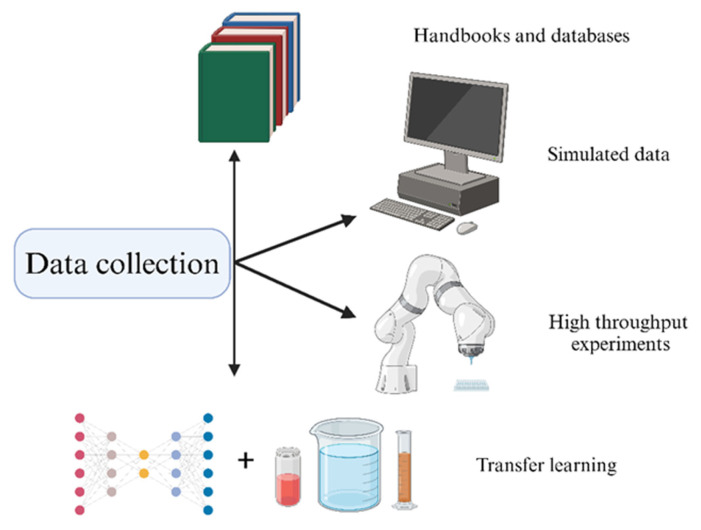
Data collection methods.

A third approach to data acquisition lies between simulated data and experimental data. Through transfer learning, a model pre-trained on a large simulated dataset can be fine-tuned using a smaller experimental dataset. This hybrid approach effectively addresses concerns related to error propagation while concurrently minimizing the quantity of required experimental data [72]. However, it is important to note that the number of experiments needed is still greater than what is typically obtained during a research endeavour [44]. This highlights the ongoing challenge of balancing data efficiency and accuracy in the modeling process. For additional insights into transfer learning, Zhuang et al. provide detailed information and summarizes over 40 representative transfer learning approaches [73]. Figure 3 illustrates the data collection methods.

Despite significant efforts in creating numerous databases, the need for comprehensive data sharing within the scientific community remains crucial. This involves ensuring data homogenization and enrichment with relevant chemical and physical properties. Implementing benchmarks or standardized testing approaches emerges as a viable strategy for achieving comparable data standards.

### 3.2. Data Translation

The properties of materials are intricately dependent on their atomic structures, making the computational representation of chemical structures a challenging task. To ensure the effectiveness of AI methods, the machine-readable representations of a material’s structure must meet specific criteria [74]: (a) the similarity or dissimilarity between two data points should accurately reflect the similarity or dissimilarity between the underlying structures they represent, (b) the representation should be broadly applicable across the full domain of materials of interest, (c) it should be computationally more efficient than directly calculating the target property. Several methodologies have been developed to address this challenge. 

Atomistic geometries can be described using local, global, and topological descriptors [74]. Local descriptors characterize the chemical structure by detailing the immediate surroundings of each atom, representing the entire structure as a composition of these localized features [75]. These representations can be generated through a Voronoi tessellation of a chemical structure. In a Voronoi tessellation, space is divided into cells, each containing a single atom and encompassing the region closer to that atom than to any other. These descriptors enable the development of AI models with high predictive accuracy, while also allowing for the separate examination of distinct fragments. This feature is particularly useful when optimizing materials compositions in materials design [76]. For example, local descriptors can define chemical bonds and their lengths, which can then be used to predict the bond dissociation energy of a compound [77].

Global descriptors consider both atom types and their positions, simultaneously defining geometric and physical properties. These descriptors, inspired by the nuclear repulsion term in the molecular Hamiltonian and free energy, represent molecules as Coulomb matrices [78]. Unlike local descriptors, which focus on specific parts of a material, global descriptors represent the entire material, allowing for faster evaluation of properties. However, their main limitation is the inability to evaluate local interactions, such as surface or interfacial interactions between materials [79].

Topological descriptors are widely used to characterize structures within complex, high-dimensional datasets. When applied to the spatial arrangements of atoms in amorphous or crystalline structures, topological methods reveal inherent geometric features that provide valuable insights for downstream predictions, including phase changes, reactivity, and separations. Among these descriptors, the persistent homology method excels in identifying significant structural features that are both machine-readable and physically interpretable [80]. However, this approach is computationally expensive, requiring a careful balance between the advantages of capturing complex structural information and the computational cost of generating these features [74]. Using these descriptors, Jiang et al. [81] developed a model for predicting the properties of crystalline materials. Trained on a dataset of over 30,000 compounds, the model revealed a limitation of topological descriptors: lower prediction accuracy was observed for compounds containing less common elements.

Graph-based representations allow to represent chemical structures from structural data. This method converts a set of atoms, characterized by atomic nodes and edges that define their relative positions, into a graph. Examples of such representations include Crystal Graph Convolution Neural Networks (CGCNN) [82], MatErials Graph Networks (MEGNet) [83], and the Simplified Molecular Input Line System (SMILES) [84].

CGCNN has demonstrated strong predictive performance for material properties such as shear modulus, as well as atomic-scale properties like formation energy [82]. However, the model does not account for global state descriptors, such as temperature, that are crucial for predicting state-dependent properties like free energy. To overcome this limitation, the MEGNet model [83] was developed, incorporating such inputs and extending the model’s application from crystals to molecules. 

Similarly, SMILES was developed to describe molecular structures of polymers as graphs [84]. However, a major limitation of SMILES lies in its inability to represent the stochastic nature of polymers. The introduction of BigSMILES aimed to address this issue, applying the SMILES syntax to repeated units and including additional bonding descriptors to accurately represent complex polymer structures [85].

Despite their advantages, graph-based representations still face challenges—in particular, their dependence on known atomic positions and their limited ability to predict molecular distortions [74]. Accurately capturing the dynamic behaviour of molecules often requires computationally expensive simulations to model structural distortions and their impact on material properties.

Stoichiometry-based representations rely on well-established atomic information, such as atomic radii, which are readily available from databases [86]. This approach makes AI methodologies more accessible to non-experts, especially compared to methods that require detailed structural data. However, it produces only a single prediction for each stoichiometry, even when multiple synthesizable structures may exist [74]. In summary, stoichiometry-based representations offer a practical solution for non-experts, particularly when atomic-level structural information is unavailable or when conducting a broad exploration of various chemical compositions.

For a more comprehensive overview of data descriptors for materials, we recommend referring to the cited review [74,87] or exploring the DScribe database [88], which provides common descriptors for materials along with tutorials for their use.

### 3.3. Model Selection

Selecting the appropriate AI model for a specific task can be challenging due to the wide range of models available. Because of the intrinsic complexity of different problems and the unique characteristics of each AI approach, certain models are better suited to specific scenarios. In biomaterials science, the main AI applications can be broadly categorized into property prediction and material design, with the latter representing an inverse design problem.

Property prediction is likely the most extensively explored task, as it is generally less complex than inverse design and can produce reliable results even with relatively small datasets [44]. Supervised regression models are commonly used to predict well-characterized properties, such as thermal behaviour, while graph-based models are particularly well-suited for graph-structured data formats (e.g., SMILES notation) [89]. Additionally, when large datasets are available, deep learning models can be effectively employed to predict material properties [90].

In contrast, material design tasks often rely on deep learning approaches, which are typically data-hungry and require large, high-quality datasets [32]. In particular, generative models, which approximate probability distributions, represent a promising approach for material discovery, although they may generate unstable or chemically infeasible compounds [91,92,93]. To address such limitations, emerging strategies such as physics-informed neural networks (PINNs) offer a compelling alternative. By embedding physical laws directly into the training process, PINNs help reduce data requirements and enhance model generalizability [94,95].

Table 3 summarizes the categories of models commonly used for property prediction and material design, along with a brief description of their data requirements and limitations. It is important to note that the size and format of the dataset should guide researchers in selecting the appropriate AI model.

## 4. Machine Learning for Biomaterials

Biomaterials, as previously defined, encompass a broad category of materials and implants engineered to interact with biological systems for medical purposes. These materials, which can be either natural or synthetic, are used across various medical fields, including diagnostic devices, dental and orthopaedic implants, drug delivery systems, and regenerative medicine. Their design aims to fulfil specific functions when in contact with biological fluids, living tissues, and organs. To achieve this, biomaterials must exhibit essential properties, such as biocompatibility, hemocompatibility (the ability to interface with blood without eliciting adverse reactions), bioactivity (the capacity to form stable chemical bonds with living tissues), as well as adequate mechanical strength and tailored degradation characteristics suited to their intended in vivo application.

To effectively design biomaterials, it is crucial to establish well-defined physical, chemical, or biological properties as input parameters, providing the basis for predicting the corresponding output properties. A clear example of this is the identification of the optimal material for bone scaffolding, as studied by Javaid et al. [98]. First, the mechanical properties of natural cortical and cancellous bone were considered in selecting the synthetic biomaterials. These properties were then used to develop an AI model. The results of their study revealed that brushite and titanium alloy emerged as the most suitable materials for mimicking cancellous and cortical bones, respectively. Conversely, polycaprolactone (PCL) and chitosan were identified as the least suitable materials for replicating cancellous and cortical bones, respectively. Furthermore, AI methodologies can be harnessed to predict the mechanical properties of devices manufactured with various patterns, as demonstrated by Agarwal et al. in their investigation of polylactic acid (PLA)-based bone screws produced via AM technique [99].

### 4.1. Ceramics and Bioactive Glasses

Ceramics find extensive application in the field of medicine owing to their remarkable biocompatibility and superior mechanical properties, particularly in wear applications. Among these ceramics, zirconia stands out for its widespread use in dentistry, thanks in part to its colour, which closely matches that of natural teeth. Therefore, in aesthetic dentistry, achieving a perfect colour match between ceramic restorations and surrounding teeth is highly challenging, due to the variable clinical situation. To address this challenge, Yang et al. [100] successfully developed a regression model based on three categories of input: material types, background colours and thicknesses. As a consequence, a large dataset of 720 samples was obtained, which was subsequently partitioned into training and validation sets at an 8:2 ratio. The model’s primary outputs—colour difference and lightness difference—were carefully chosen to gauge its efficacy. The resulting model demonstrated impressive predictive capabilities. However, it is important to note, as acknowledged by the authors, that its utility lies in serving as a time-saving guidance tool rather than a substitute for the expertise of dental professionals. Ultimately, the application of knowledge and clinical expertise by professionals remains primary in making the final determination. 

In orthopaedics, hydroxyapatite (HA) is among the most commonly used ceramics, given its status as one of the main components of natural bone [101]. Trying to design HA scaffolds with mechanical, physical, and microarchitectural properties resembling those of bone, Ibrahimi et al. [102] explored scaffold architectures based on Triply Periodic Minimal Surfaces topology. Initially, they employed an ML model to generate a set of scaffolds with the desired architecture. Then, the mechanical, physical, and morphometric properties of the generated scaffolds were assessed using the finite element method (FEM). The acquired properties served as a dataset for another ML model, which predicted the optimal topology parameters to achieve specific properties. Simulation tests on the optimized scaffold demonstrated properties comparable to trabecular bone. However, it is worth noting that the feasibility of this structure with current technologies remains unverified. Additionally, the model did not consider the possible presence of defects resulting from the manufacturing process. Further studies on these aspects are necessary to ascertain the reliability of the model.

Bioceramics can also exhibit bioactive and bioresorbable behaviours within a biological system. Furthermore, incorporating doping agents into ceramic compositions can enhance or introduce certain therapeutic properties. Common examples of such behaviours include HA, β-tricalcium phosphate (β-TCP), and bioactive glass (BG). These materials are often utilized as fillers, for example, in bone injuries, to aid in the healing process. To explore the relationship between chemical compositions and physical behaviours in HA nanopowders, Yu et al. [103] developed two AI models, an ANN and a genetic programming model, using their own experimental dataset. The robustness of these predictive models was subsequently validated using a genetic programming approach. The results showed good accuracy of the models in predicting the crystallite size, micro-strain, and grain boundary volume fraction of HA nanopowders based on chemical compositions, with an *R*^2^ > 0.8 for each variable. Evaluating the correlations between chemical compositions and physical properties is fundamental also for BG development. However, measuring these properties can pose challenges due to the various techniques involved in characterization. In response to this challenge, Cassar et al. [104] devised a model by gathering data from the SciGlass database [55], aiming to predict parameters such as glass transition temperature, liquidus temperature, Young’s modulus, thermal expansion coefficient, refractive index, and Abbe number. Specifically, they compared three different AI algorithms. The results showcased high predictive accuracy across all models, attributed to the high amount of data utilized. Nonetheless, it is worth noting that these predictions exhibit higher errors for glasses containing chemical elements with limited representation in the training dataset.

Understanding ceramic bioactivity and the resulting bone formation from a biomaterial graft is non-trivial, typically necessitating expensive and time-consuming in vivo tests. To address this challenge, numerous studies are focusing on predicting bone formation following graft procedures. Wu et al. [105] have developed an ANN model that correlates the outcomes of an animal study involving scaffold treatment for a major segmental defect in sheep tibia. Both bone regeneration outcomes and simulated data were utilized as training data for this model. Remarkably, the model successfully predicts bone remodelling at 12 months following the application of a Sr–Hardystonite–Gahnite scaffold in a large segmental defect of a sheep tibia. With a similar objective, Motojima et al. [106] built a ML model capable of predicting bone formation rates, material properties, crystalline phases, and functional groups based on test settings. This model was also designed to aid in the formulation of experimental conditions for novel bioceramics. Remarkably, the model successfully facilitated the design of new biomaterials with enhanced bone regeneration capabilities. However, it is important to note that no experiments were conducted to validate the model initially. Subsequent to this limitation, a follow-up study by the same research group addressed this gap by validating the predicted bone regeneration rate through experimental tests conducted on porcine tibiae [107]. In particular, they constructed two ML models – one mapping fabrication conditions to scaffold properties, and another predicting in vivo bone-forming ability from those properties. An inverse design analysis proposed fabrication recipes for target bone formation; the actual bone formation in 12-week porcine implant tests fell within the predicted error range.

Similarly, Lin et al. [108] conducted a study to assess the accuracy of ML models in predicting the osteoinductivity of biomaterials, using a literature-derived database spanning three decades. To address challenges related to limited or incomplete data, a data enhancement strategy was employed. The resulting model was then used to guide the experimental design of a biphasic calcium phosphate scaffold, which was tested in an in vivo study on beagles. The study demonstrated that the extreme gradient boosting algorithm (XGBoost) outperformed other models in predictive accuracy and enabled the design of a scaffold that exhibited enhanced osteogenesis compared to the average outcomes reported in the literature. These studies demonstrate how AI predictions of bio-ceramic performance (e.g., osteogenesis) can be confirmed by animal studies, effectively bridging the gap between computational design and biological reality.

The resorbability of BG is attributed to their solubility in aqueous environments, a characteristic closely linked to the chemical composition of the specific BG. Numerous studies have explored the variations in BG compositions to optimize their degradation behaviour. Among these efforts, Brauer et al. [61] attempted to address this challenge using an ANN model. Their study focused on the dissolution of phosphate-based BG in deionized water, aiming to model the solubility behaviour of these glasses. While the study highlights the potential of this innovative approach, the limited number of experiments conducted may have constrained the accuracy of the model. This limitation was overcome by Han et al. [109], who developed a model based on a comprehensive dataset comprising over 1300 experimental observations. Their model was specifically trained to predict the dissolution behaviour of four distinct types of BG: sodium borosilicate, sodium aluminoborate, sodium aluminoborosilicate, and sodium boro-phosphosilicate. Each dataset entry included ten inputs (such as glass composition, initial solvent pH, temperature, system surface area-to-volume ratio, and time) and one output (expressing the degree of glass dissolution as normalized mass loss of critical ions). The results showed the model’s exceptional predictive capabilities. However, as noted by the authors, a broader and more diverse database is necessary to enable the model to engage in optimization processes, such as predicting the requisite BG composition to achieve a desired dissolution rate.

Thanks to their dissolution in biological environments, BG can exploit their therapeutic effects. By manipulating their chemical composition, it is possible to give various desirable traits, including antibacterial activity. Various elements, such as Ag and Cu, have been explored for their antibacterial potential [110,111]. However, achieving optimal chemical compositions can be time-consuming due to the multitude of variables involved. In an effort to simplify this research, Echezarreta and Landin [112] developed an algorithm capable of predicting the antibacterial effects of BGs. Following an extensive literature review to identify critical variables influencing antibacterial effects, they employed neuro-fuzzy logic to reduce the complexity, focusing on key variables: SiO_2_ concentration (*w*/*w*%), CaO concentration (*w*/*w*%), BG concentrations (mg/mL), bacterial species, and the pH recorded post-bacterial cultivation with BG. Fuzzy systems, like the one employed in this study, are well-suited for tackling problems with unclear or ambiguous variable relationships. By utilizing a rule-based approach, fuzzy systems can integrate expert knowledge and effectively handle uncertainty [113]. Their findings revealed that antibacterial activity primarily depends on the release of alkaline ions into the solution, resulting in a rise in pH levels. Notably, differences in BG antibacterial efficacy across different bacterial species are primarily attributable to the composition of BG, particularly its calcium ion content.

Due to their aforementioned properties, BGs can be used in orthopaedics for bone tissue restoration. Specifically, AM is frequently employed to create porous structures, typically referred to as scaffolds, which aim to mimic the architecture of natural bone tissue [10]. However, optimizing scaffolds poses challenges due to the complexity of bone structure. In addressing this issue, AI techniques are applicable for optimizing the design of AM bone scaffolds, offering a promising avenue for enhancing scaffold performance and functionality. In a pioneering effort, Wu et al. [114] implemented a mechano-biological optimization strategy for 3D printed bioactive glass-ceramic scaffolds with cost-effective evaluation methods. The study demonstrated the design of scaffolds tailored for critical size segmental bone defects in sheep tibiae, employing a lithography-based ceramic manufacturing technique to fabricate the optimized scaffolds. Three distinct structures were chosen as candidate unit cell structures for optimization. In this investigation, the Young’s modulus associated with each unit cell was treated as input, while the locally varying micro-strain component linked with each unit cell served as the output. The results demonstrated that the optimized functionally graded scaffolds outperformed conventional empirical designs, resulting in enhanced bone formation.

### 4.2. Polymers

Polymers are extensively used in tissue engineering thanks to their adaptability, optimal biocompatibility, and good mechanical properties. Currently, synthetic polymers serve as the preferred choice due to their higher mechanical properties and reduced allergic response compared to natural polymers [115]. In both cases, mechanical, chemical, and biological properties can be adjusted by combining different monomers or modifying their ratios. The resulting polymer, known as a copolymer, exhibits a combination of properties derived from its constituent monomers [116]. However, predicting copolymer properties can be challenging due to the numerous variables. Good results have been obtained with an AI model that used a weighted average of two or more homopolymer fingerprints to generate a copolymer fingerprint, and predict the glass transition temperature (*T_g_*), melting temperature (*T_m_*), and degradation temperature (*T_d_*) of copolymers with an *R*^2^ value of 0.94 [117]. These characteristic temperatures are fundamental to characterizing a polymer, since they are indicators of structural properties, processability and thermal stability. Additionally, the same group compared the accuracy of single-task and multi-task models in predicting 36 polymer properties. Results revealed that single-task model are more sensitive to data sparsity and missing data, with normalized root mean squared error (*RMSE*) up to 1. On the other hand, the multi-task approach addressed the challenge of limited data availability by identifying inter-property correlations and required less training time compared to many single-task models [63].

Dataset size is a common issue in AI approaches. An alternative for addressing this challenge in polymer science is the use of simulated data based on a large dataset of molecular building blocks. Utilizing this solution along with a variational autoencoder (VAE) model, Batra et al. [118] designed novel polymers with high thermal and electrical requirements. Moreover, the study highlighted the versatility of this approach for predicting numerous properties. Similarly, polymers can be designed using a genetic algorithm (GA), a model based on the natural selection process. This method visualizes a polymer as a sequence of interconnected building blocks that define its properties and can be rearranged to achieve different characteristics. Although this approach requires fewer tuning parameters compared to other generative models, it is highly influenced by the dataset, the mutation rate, and the available building blocks [119].

AI models exhibit a notable capability to accurately predict the mechanical properties of materials. This attribute becomes particularly advantageous in scenarios where mechanical assessments pose challenges, such as with hydrogels. Hydrogels represent a category of polymers extensively utilized in diverse areas of tissue engineering, notably in the regeneration of soft tissues like skin and muscle. These materials are defined by their structure as networks of water-soluble polymers, which can be crosslinked either physically or chemically to form a gel. However, one of the primary drawbacks associated with hydrogels is their inherently low mechanical properties, particularly when compared to other biomaterials [103]. To predict the mechanical properties of hydrogels, a model was developed to examine the effects of alginate dialdehyde (ADA) content, cross-linker content, pore size, and BG content on the stiffness of ADA-gelatine hydrogels using the extreme gradient boosting ML algorithm. The model showed high predictive accuracy and appeared to fit the data well. However, the absolute errors between the predicted and observed values remained relatively high. This discrepancy may be attributed to the small dataset, based on literature data, which likely led to overfitting, as noted by the authors [62].

An analogous study focused on Polyvinyl alcohol (PVA)/Gelatine porous hydrogels [120]. Leveraging over 30.000 data points obtained from mechanical tests, the model employed input parameters including the concentrations of PVA, Gelatine, and cross-linker, alongside the duration of the tests. The resultant stress and strain values in a uniaxial compressive test constituted the model’s output. Demonstrating high accuracy across all three regimes—linear elastic, plateau, and densification—the predictive model proved to be highly accurate. Similarly, Das et al. [121] developed a model to predict the porosity of alginate gel scaffolds. The model was trained with over 100 samples with known porosities and considered viscosity, contact angle, and surface tension as inputs. Subsequently, the model was employed to forecast the optimal parameters for achieving maximum porosity, which were then utilized to fabricate an alginate gel scaffold, resulting in an apparent porosity closely aligned with the predicted values. Notably, the model demonstrated considerable potential, particularly due to its incorporation of the contact angle, a parameter greatly influenced by surfactant content, as an input.

Cytotoxicity—defined as the property of being toxic to cells—is a pivotal parameter in assessing the safety of materials for biological applications. Nevertheless, conducting cytotoxicity tests requires significant time and expertise. Thus, the ability to predict the cytotoxic behaviour of materials holds considerable promise. In response to this challenge, Xu et al. [122] developed a decision tree algorithm to forecast the cytotoxicity of micro- and nano-plastics with an accuracy of 95%. Their dataset was compiled through a comprehensive literature review on the subject. Their findings revealed that key factors influencing cell viability include the plastic source, zeta potential, exposure concentration, and the biological model representing various cell types. This suggests that cytotoxicity manifests as an intrinsic property of micro- and nano-plastics within specific cell lines under defined experimental conditions. Notably, the study did not identify a discernible relationship between particle size and toxic effects.

Another important biological parameter is cell adhesion and proliferation. Through the use of a computational model Hao et al. [123] optimized a mixture of polyethylene glycol (PEG) and arginine-glutamic acid-aspartic acid-valine peptide to obtain high endothelial cell selectivity against smooth muscle cells. The dataset was derived from numerous experiments, and the resulting model (ResNet50V2) achieved an accuracy of over 95%. Additionally, the optimal composition was used as a surface coating formula for implantable cardiovascular devices made of nickel-titanium, demonstrating successful endothelialisation of the surface.

An inflammatory response is a typical phenomenon that follows the implantation of grafts or medical devices, as our bodies recognize foreign materials and attempt to reject them. The general strategy to maximize engraftment rates is to minimize the host immune response through anti-inflammatory therapies [124]. A contribution to minimizing the inflammatory process can be made by using polymers with anti-inflammatory properties. To predict the immunomodulatory potential of polymers, two models—K-nearest neighbours and Naïve Bayes—were compared by Akkache et al. [125]. The models used cellular assays of 50 polymers on murine macrophages as training data for immunomodulatory behaviour. The secretion of nitric oxide and tumour necrosis factor alpha by macrophages was considered as input markers for the inflammatory process. The best accuracy was achieved by the K-nearest neighbours model; however, the limited data and the oversimplified system considered reduce the model to an initial decision-making step rather than an efficient classifier.

In another study, Gong et al. [126] applied AI models to a dataset of poly(beta-amino ester) (PBAE) gene-delivery polymers. The models predicted which polymer compositions would yield high in vitro transfection efficiency. Synthesized candidate polymers were tested in macrophage and liver cell cultures, and predicted transfection levels correlated strongly with experimental values (Spearman’s *ρ* ≈ 0.57–0.66). Notably, model explainability analysis identified tuneable parameters, like hydrophobicity and carbon atom count, that significantly influence gene delivery performance. These insights offer valuable guidance for the rational design of next-generation polymeric gene delivery systems.

Polymers can also exhibit antimicrobial properties, as demonstrated in numerous studies [127]. In order to easily identify peptides with high antimicrobial properties, Huang et al. [128] developed a model that mines billions of peptide sequences for peptides composed of 6–9 amino acids with potential antimicrobial properties. The identified peptides showed excellent results against a wide range of multi-drug-resistant pathogens. Further confirmation of the model’s reliability was obtained through in vivo tests in mice with bacterial pneumonia, which showed effects comparable to penicillin.

The high moldability of polymers makes them suitable for numerous manufacturing techniques. Among these, electrospinning is a common technique in tissue engineering to produce fibrous scaffolds for wound healing [129]. The influence of process parameters on mechanical properties can be evaluated using AI models. This approach was successfully applied to predict the tensile strength and suture retention of PCL using an ANN. Notably, the model exhibited a negligible difference between experimental and predicted data, with deviations of less than 2.8% for suture retention and under 0.4% for tensile strength [130]. Alternatively, evaluating the effects of electrospinning parameters on the orientation, angle and diameter of the resulting fibres can represent a method for process optimization. In particular, a convolutional neural network has demonstrated higher predictability compared to other regression models when faced with a limited amount of data. Notably, to mitigate model overfitting caused by the small data size, the authors employed oversampling, hyperparameter optimization, and k-fold cross-validation during models’ development and evaluation [131].

Similarly, in a comparative study on the process optimization of fused deposition modelling using a dataset derived from the literature, XGBoost emerged as the best predictor, achieving an *R*^2^ above 0.94 [132]. Further refinement of the model can be obtained by the use of Shapley Additive exPlanation (SHAP), as demonstrated by another study, which achieved an *R*^2^ of 0.976. However, the hierarchical importance of the printing parameters differed between the models with and without the use of SHAP, highlighting how the choice of analysis technique influences the output [133].

Optimization has also been a focus in bioprinting, as demonstrated by Mohammadrezaei et al. [134]. In this study, the authors collected both experimental data and bibliographic data, resulting in a dataset of 591 data points. The chosen model was a regression neural network, built to predict cell viability for gelatine- and alginate-based bioinks, and to identify the most critical parameters. However, the model achieved an *R*^2^ of 0.71, highlighting the need for additional data to refine it. One possible way to increase the experimental data for process optimization could be to use a design of experiments (DoE) approach [135].

It is worth emphasizing that the methods discussed earlier represent just a fraction of the diverse techniques available in polymer science. This is particularly evident when considering that the methods employed in drug discovery can also be effectively applied in polymer science, given the shared use of small molecules in their synthesis [136]. For an in-depth exploration of machine learning methods applied in polymer science, we recommend consulting the comprehensive reviews provided [37,44,136].

### 4.3. Metals

Metals find extensive use in applications requiring robust mechanical properties, particularly in orthopaedics and dentistry. Titanium and its alloys (Ti alloys) are among the most widely used materials in biomedical applications. Ti alloys offer numerous advantages, including high specific strength, exceptional corrosion resistance in biological environments, and favourable biocompatibility. Additionally, they provide a balance between low elastic modulus and high strength, making them particularly desirable. This characteristic is crucial, as a material with an elastic modulus comparable to that of bone can significantly reduce the risks of stress shielding and potential implant failure [137]. However, despite these benefits, the discrepancy between the elastic modulus of Ti alloys (approximately 50–120 GPa) and that of cortical bone (approximately 30 GPa) still does not fully prevent stress shielding [138]. Among these alloys, Ti-6Al-4V remains the most widely used due to its relatively low modulus compared to other alloys. Nevertheless, it faces a significant challenge regarding the release of vanadium and aluminium, which are implicated in various diseases such as Alzheimer’s [139]. Consequently, extensive research efforts are focused on identifying alternative alloy compositions with reduced biological risks and elastic moduli more closely matched to bone.

To obtain a titanium alloy with an elastic modulus comparable to that of bone, Wu et al. [140] developed an algorithm to predict compositions meeting this criterion. Their approach was based on the premise that a Ti alloy’s low elastic modulus could be achieved by stabilizing the *β* phase, which correlates with the martensitic transformation start temperature (*Ms*). By using the alloying elements’ content (in *wt*%) as input and elastic modulus and *Ms* as outputs, they identified the Ti-12Nb-12Zr-12Sn alloy as having the desired properties. Subsequent testing confirmed its high tensile strength, satisfactory ductility, and biocompatibility. The same topic was recently explored using 254 quenched *β*-Ti alloy compositions from the literature, comparing different AI models. The findings highlighted the need for complex prediction models to understand the relationship between alloy compositions and elastic modulus. As a result, the XGBoost model exhibited the highest accuracy, with an *R*^2^ of 0.962 [141].

Similarly, Jha et al. [142] investigated the same alloying elements, Nb, Zr and Sn, to produce a fully stabilized *β* phase Ti alloy. They employed a hybrid approach, combining Calculation of Phase Diagram (CALPHAD) techniques with AI models to predict the concentrations of metastable phases for novel compositions and the optimal temperature for maximizing the stability of the *β* phase. Their study generated predictions for five compositions expected to satisfy their criteria for *β* phase stability. Furthermore, a subsequent numerical study [143] confirmed the efficacy of Nb, Zr, and Sn as key alloying elements to decrease the elastic modulus while maintaining adequate yield strength in Ti alloys. Additionally, the study suggested a beneficial effect of Mo and V. Three different models (multiple linear regression, fuzzy inference systems, and ANN) were compared to elucidate the roles of alloying elements and processing parameters in determining these two mechanical properties. The models were trained on literature data and considered both composition and thermal treatment as input variables. The results showed the superior performance of ANN compared to the other models, with *R*^2^ above 0.88. However, it is important to note that no experimental validation was performed to confirm these predictions.

Sultana et al. [144] conducted a multi-objective study to address mechanical and biological challenges, aiming to identify an alloy with a low elastic modulus, high yield strength, improved biocompatibility, and cost-effectiveness. As expected, their findings suggested the utilization of *β*-Ti alloys, well-known for their low modulus. Specifically, the optimal solutions recommended a high content of Nb, moderate amounts of Ta, Mo, Cr and Zr, and minimal to no presence of Al and V. Pursuing a similar objective, Banu and Rani [145] used ANN models to predict tensile strength, yield strength and modulus based on alloy composition, thermomechanical processing and microstructure. Their investigation focused on Ti-xNb-yTa alloy, optimizing *x* and *y* (*wt*%) and examining the role of minor alloying agents. The simulations illustrated increased tensile strength and yield strength with higher Ta and Nb contents. Additionally, the modulus was observed to decrease with elevated levels of Ta and Nb, while the presence of Al was associated with an increased in modulus. Furthermore, adding Zr to Ti-Nb and Ti-Nb-Ta alloys further reduced the modulus, enhancing biocompatibility.

AI has been applied to predict biological responses to metal implants. In particular, the macrophage immune response when in contact with the titanium surface was studied by Chen et al. [64], comparing different AI models. The models—RF, XGBoost, SVM and multilayer perceptron (MLP)—were trained on literature data and considered both surface properties (e.g., roughness) and material–cell interaction (e.g., cell seeding density) as input features, with pro- and anti-inflammatory cytokine levels as target outputs. Notably, RF, XGBoost and MLP showed an *R*^2^ > 0.7; however, only the MLP model was selected for in vitro verification due to its lower mean absolute percentage error. Results showed that, despite some discrepancies between predicted and experimental values, the overall trends were consistent, demonstrating the potential of the model. The authors attributed these discrepancies to four main factors: the limited training dataset, which excluded potentially relevant features such as elemental composition; variability and possible errors in the experimental data; range constraints during data input that failed to capture reported variability; and heterogeneous distribution of the literature-derived data.

Similarly, the osteogenic response to modified titanium surfaces was studied using a DL approach. The model leveraged early-stage osteoblast morphology to predict alkaline phosphatase activity, a key marker of osteogenesis. It demonstrated excellent predictive performance, which was further validated by in vitro experiments [146]. 

Another class of metals is represented by bulk metallic glasses (BMGs). These materials have gained significant attention in recent decades due to their unique amorphous atomic structures. BMGs typically exhibit high strength, lower Young’s modulus, improved wear resistance, good fatigue endurance, and excellent corrosion resistance [147]. To identify new BMG compositions, Douest et al. [65] developed two predictive AI models for the Ti-Zr-Cu-Pd system. Data were collected from a literature review, resulting in a dataset of 643 BMGs. The first model was trained on the atomic percentages of the constituent elements, while the second model utilized material and chemical properties associated with each alloy. In both cases, the output variables considered were the maximum rod diameter (*D*_max_) at which a fully amorphous structure is preserved and the supercooled liquid region (Δ*T_x_*). The obtained compositions were then classified to maximize *D*_max_ and ΔT_x_ while minimizing the Cu content. Only the most promising candidate was produced to verify the accuracy of the models, revealing discrepancies between predicted and experimental outputs. The limited predictability of the models may be attributed to the small and relatively homogeneous dataset, as well as the restricted number of experimental trials conducted on the predicted compositions.

### 4.4. Composites

A composite material is created by combining two materials with distinct physical and chemical properties, where the primary phase is referred to as the matrix, and the secondary phase is known as the filler. In biomaterial applications, composites take advantage of the combined advantageous properties of multiple materials simultaneously, making them highly versatile and valuable in various medical fields.

In orthopaedics, ultra-high molecular weight polyethylene (UHMWPE) is a widely used polymer known for its excellent mechanical and tribological properties. To further enhance these properties, Vinoth et al. [148] incorporated carbon fibers into UHMWPE and utilized a multi-objective approach to optimize the composite composition. Their study considered both nano and micro carbon fibres as reinforcement, with a total of 16 input parameters, including the weight percentage and particle size of the nano/micro particles. The developed ANN models were used to minimize the output factors of the coefficient of friction and specific wear rate, while simultaneously maximizing the elastic modulus, hardness, and ultimate tensile strength. Notably, the study highlighted the differences between single-objective and multi-objective optimization approaches, emphasizing the importance of considering multiple factors simultaneously when optimizing composite materials.

As we mentioned in the previous paragraphs, hydrogels are a class of polymers known for their low mechanical properties [103]. One viable strategy for enhancing their mechanical properties is to create composites in which hydrogels serve as the matrix. Conventional approaches for determining the mechanical properties of these composites typically involve experiments or FEM. However, both approaches have drawbacks, such as being time-consuming and computationally expensive. Combining FEM and AI can address these issues, as shown in different studies [149,150]. In fact, Gholami et al. [150] demonstrated the high accuracy of this approach by comparing two deep neural network models, ResNet and AlexNet. Their study considered a dataset of 9000 generated microstructural images with different particle shapes, along with their Young’s modulus and Poisson’s ratio computed by FEM analysis. The models exhibited high accuracy in predicting the mechanical properties of collagen hydrogel reinforced with BG particles. Notably, the AlexNet model demonstrated the highest accuracy, with an *R*^2^ > 0.92 for all particle shapes. Similarly, the combination of FEM analysis and deep neural networks has proven highly effective in extracting the elastic properties of the constituent phases in composites. This approach is particularly promising for evaluating the mechanical properties of cells within real tissues, as in vitro analyses on isolated (non-embedded) cells often yield results that differ significantly from those of embedded cells, and are typically challenging and unreliable [151]. Moreover, the same research group has demonstrated that this method is also feasible for predicting the surface elastic properties of materials from feature images that describe the mechanical and geometric characteristics of surface microstructures, further highlighting the versatility of the approach [152].

Alternatively, the combination of AI and response surface methodology (RSM) has also proven efficient, as shown in a study on composite hydrogels containing heparinized ZnO nanoparticles [153]. In this study, different models were compared to predict the degree of swelling and the water vapor transmission rate as the composition of the composite hydrogel varied. After determining the best model, the results were used to graphically optimize the composition using RSM. The optimal composition was then produced and characterized, revealing small discrepancies between the actual and predicted properties. A contrasting approach was taken in the study by Shera et al. [154], which compared the accuracy of ANN and RSM in predicting the effects of composition, porosity, and swelling on the drug release of chloramphenicol in a silk fibroin–xanthan gum composite scaffold. The experimental tests demonstrated the higher accuracy of ANN compared to RSM, highlighting the success of ANN in predicting non-linear relationships. A similar trend was observed in a study by Boztepe et al. [155], which investigated drug release behaviour from highly swellable, pH- and temperature-responsive hydrogels synthesized via interpenetrating polymer network techniques using free radical polymerization in the presence of microgels and a pore-forming agent. ANN, SVM, and support vector regression (SVR) models were compared for predicting doxorubicin release under varying pH and temperature conditions. Using a dataset of 540 experimental observations for model training and validation, the results again demonstrated the superior accuracy of ANN in capturing complex, non-linear relationships.

The use of AI models can be extended also to nanocomposites. Zhu et al. [156] demonstrated the power of combining high-throughput phase-field simulations with ML to design Ti-Nb nanocomposite alloys with exceptional mechanical properties, including ultralow Young’s modulus, linear super elasticity, and nearly zero hysteresis. Using an ANN for multi-objective optimization, the study identified ideal microstructural configurations based on Nb concentration, nanofiller volume fraction, and other parameters. This approach led to the discovery of a nanocomposite with properties suitable for orthopaedic implants, addressing key challenges like stress shielding and hysteresis-related inefficiencies in shape memory alloys. However, a key limitation of the study lies in the absence of experimental synthesis and mechanical testing of the proposed alloy, which is necessary to confirm the reliability of the computational predictions. A similar effort in nanocomposite optimization is seen in the work of Wang et al. [157], where ANNs were used to predict various physical and mechanical properties of PVA-based scaffolds, including pore size, porosity, compressive strength, elastic modulus, and water absorption. These predictions were made based on the weight percentages of nano-hydroxyapatite and magnetic nanoparticles incorporated into the PVA matrix. Both studies reported a *R*^2^ greater than 0.96, highlighting the high predictive accuracy of ANN models and reinforcing the potential of ML-driven approaches in optimizing biomechanical compatibility in nanocomposites.

Composite materials can also be used as bioinks for scaffold production. An early attempt to predict the printability of biomaterial ink formulations for AM scaffold production involved comparing decision trees, random forests, and DL models [158]. The models were trained on experimental data from 210 formulations derived from 16 distinct biomaterials. The results indicated that the random forests model had the best accuracy; however, the DL model exhibited a higher recall, meaning it was less likely to miss printable formulations. Notably, all models displayed signs of overfitting.

Similarly, Hashemi et al. optimized a chitosan-agarose-gelatine ink [159]. The printing parameters were refined using Bayesian optimization based on previous work [160], and a transfer learning approach was applied to include ink composition among the input variables. This strategy proved to be beneficial, as it identified the optimal parameters in fewer trials compared to those typically required by experienced users. Analogous studies for the optimization of bioinks can be found in the works of Lee et al. [161] and in the review article of Ramesh et al. [162].

## 5. Challenges and Future Perspectives

The role of AI in biomaterials science presents numerous challenges and opportunities. Computational models enable the prediction of many properties—both biological and non-biological—that would otherwise require significant resources to evaluate. However, achieving reliable and accurate predictions, particularly for properties that are less commonly evaluated (e.g., bioactivity, cytotoxicity, etc.), requires large volumes of high-quality, standardized data for model training and validation. In fact, the primary cause of low prediction accuracy is often the use of small, sparse, or heterogeneous datasets. An ideal solution would be the standardization of property evaluations and comprehensive data sharing, in agreement with the principles of FAIR (Findable, Accessible, Interoperable, Reusable) [163]. Nonetheless, a practical approach to address this issue could involve data mining from the literature or artificially increasing dataset size through data augmentation techniques [164]. For instance, PINNs embed known physical laws (e.g., diffusion or reaction kinetics) into the learning process, enabling accurate predictions even with limited data. Generative models like GANs or diffusion models can augment datasets by synthesizing realistic new examples. Recent work like MatterGen uses a diffusion-based model to produce novel, stable materials given target properties [93]. Such models can, in effect, simulate a vast space of biomaterial designs to supplement experimental data. However, it is crucial to ensure that the dataset used is as clean as possible through data preprocessing, normalization, and outlier detection to avoid redundant or noisy data, which could negatively impact model performance.

Additionally, the use of different AI models may lead to different results, and the black-box nature of these models limits our understanding of the principles behind the prediction process. This highlights the need for experimental validation or for more transparent techniques, such as the Shapley Additive Explanation, as suggested by FDA guidelines for AI-based medical products [165]. Moreover, the properties of biomaterials are often strongly linked to the analytical methods and processing conditions, which complicates the adaptation of data to different contexts. A potential solution to this challenge would be to use models that integrate traditional computational methods, such as finite element analysis, or domain knowledge, such as physical or chemical laws, to enhance the interpretability and reliability of the models.

A concrete proposal for integrating AI with regulatory standards and experimental validation could combine these approaches. First, data acquisition and preprocessing should be transparent and well-documented to ensure reproducibility. Second, models should meet clear performance metrics, such as high *R*^2^, low *RMSE*, and the use of explainable AI techniques (e.g., Shapley Additive Explanation) to enhance interpretability. Third, validation should follow a multi-stage protocol (in silico, in vitro, and in vivo), and, when feasible, include interlaboratory testing to assess robustness. In addition, the use of benchmark datasets would facilitate comparison across studies, and a standardized datasheet or reporting template could help users quickly evaluate model quality and compliance.

## 6. Conclusions

AI and its derivatives are currently a hot topic, as highlighted by the increasing number of studies that use this approach. Indeed, AI models represent powerful tools that can potentially improve the quality of research, reduce time and resource waste, and find alternative patterns that are difficult for human knowledge to identify. Nonetheless, the findings presented in this study suggest that this technology is still immature, often leaving room for reasonable doubt regarding the predicted results. Consequently, it is imperative to recognize AI as an exceptional complementary resource rather than a replacement for traditional research and studies. Therefore, critical supervision of the obtained results is always highly recommended to ensure the accuracy and validity of AI-assisted findings. Embedding FAIR data practices and adhering to emerging AI guidance will help translate biomaterial AI/ML models into safe, effective clinical technologies.

## Figures and Tables

**Table 1:** Pros and cons of traditional and AI algorithms.

Traditional Algorithms		Artificial Intelligence Algorithms	
Pros	Cons	Pros	Cons
Transparent process	Limited versatility	Free from assumption	Lack of interpretability of the process
High efficiency for well-defined problems	Human-dependency	High adaptability and scalability	Data-dependency
Low computational requirements	-	-	High computational requirements

**Table 3:** Brief description of models commonly used in material science. Random forest (RF), support vector machine (SVM), Gaussian process regression (GPR), crystal graph convolution neural networks (CGCNN), materials graph networks (MEGNet), artificial neural networks (ANN), convolutional neural network (CNN), generative adversarial networks (GAN), variational autoencoders (VAE).

Model	Requirements	Advantages	Limitations	Ref.
Classical ML (RF, SVM, GPR, etc.)	Effective on small to medium-sized datasets (ranging from tens to thousands of samples) when using structured, curated features.	Easier interpretation. Fast training on structured data.	Requires manual feature engineering. Limited generalization and sensitive to data redundancy.	[19,96]
Graph Neural Networks (CGCNN, MEGNet, etc.)	Require large graph-based datasets.	Can capture complex relational/structural information.	Computationally demanding. Sensitive to training quality. Poor performance on limited data.	[89]
Deep learning (ANN, CNN, etc.)	Requires large, high-dimensional datasets (e.g., images, spectra). Benefits from pre-training.	Can learn complex, non-linear relationships. Flexible input types.	Requires tuning and high compute resources. Black box. Overfitting on small data.	[32]
Generative models (GAN, VAE, etc.)	Requires large datasets (often >10^4^ samples). Typically uses composition matrices or structural encodings derived from large databases.	Can generate novel materials and expand datasets.	Generated structures may violate chemical or stability constraints. Difficult to train.	[91,92,93,97]

## Data Availability

Not applicable.

## Abbreviations

The following abbreviations are used in this manuscript:

ADA	Alginate Dialdehyde
AM	Additive Manufacturing
AI	Artificial Intelligence
ANN	Artificial Neural Network
BG	Bioactive glass
BMG	Bulk metallic glass
CALPHAD	Calculation of Phase Diagram
CNN	Convolutional neural network
CGCNN	Crystal Graph Convolution Neural Networks
DL	Deep Learning
T_d_	Degradation temperature
DoE	Design of Experiments
XGBoost	Extreme gradient boosting
FAIR	Findable, Accessible, Interoperable, Reusable
FEM	Finite element method
GPR	Gaussian process regression
GA	Genetic algorithm
GAN	Generative adversarial networks
T_g_	Glass transition temperature
HA	Hydroxyapatite
ML	Machine Learning
Ms	Martensitic transformation start temperature
MEGNet	MatErials Graph Network
D_max_	Maximum rod diameter
T_m_	Melting temperature
MLP	Multilayer perceptron
PINNs	Physics-Informed Neural Networks
PCL	Polycaprolactone
PEG	Polyethylene glycol
PLA	Polylactic acid
PVA	Polyvinyl alcohol
RF	Random forest
RSM	Response surface methodology
RMSE	Root mean square error
SHAP	Shapley Additive exPlanation
SMILES	Simplified Molecular Input Line system
SVM	Support vector machine
SVR	Support vector regression
ΔT_x_	Supercooled liquid region
Ti alloy	Titanium alloy
UHMWPE	Ultra-high molecular weight polyethylene
VAE	Variational autoencoders
β-TCP	β-tricalcium phosphate
